# Fibronectin binding protein B binds to loricrin and promotes corneocyte adhesion by *Staphylococcus aureus*

**DOI:** 10.1038/s41467-022-30271-1

**Published:** 2022-05-06

**Authors:** Thaina M. da Costa, Albertus Viljoen, Aisling M. Towell, Yves F. Dufrêne, Joan A. Geoghegan

**Affiliations:** 1 Department of Microbiology, Moyne Institute of Preventive Medicine, School of Genetics and Microbiology, Trinity College Dublin, Dublin 2, Ireland; 2 Louvain Institute of Biomolecular Science and Technology, UC Louvain, Croix du Sud, 4-5, bte L7.07.07, B-1348 Louvain-la-Neuve, Belgium; 3 Walloon Excellence in Life sciences and Biotechnology (WELBIO), Wavre, Belgium; 4 Institute of Microbiology and Infection, University of Birmingham, Edgbaston, Birmingham, B15 2TT United Kingdom

**Keywords:** Bacterial pathogenesis, Imaging techniques and agents

## Abstract

Colonisation of humans by *Staphylococcus aureus* is a major risk factor for infection, yet the bacterial and host factors involved are not fully understood. The first step during skin colonisation is adhesion of the bacteria to corneocytes in the stratum corneum where the cornified envelope protein loricrin is the main ligand for *S. aureus*. Here we report a novel loricrin-binding protein of *S. aureus*, the cell wall-anchored fibronectin binding protein B (FnBPB). Single-molecule force spectroscopy revealed both weak and ultra-strong (2 nN) binding of FnBPB to loricrin and that mechanical stress enhanced the strength of these bonds. Treatment with a peptide derived from fibrinogen decreased the frequency of strong interactions, suggesting that both ligands bind to overlapping sites within FnBPB. Finally, we show that FnBPB promotes adhesion to human corneocytes by binding strongly to loricrin, highlighting the relevance of this interaction to skin colonisation.

## Abstract (web summary)

The first step during skin colonization by is its adhesion to corneocytes. Da Costa et al. show that the cell wall-anchored fibronectin binding protein B (FnBPB) of *S. aureus* binds to loricrin. Applying single cell force spectroscopy, they demonstrate that this interaction promotes adhesion of *S. aureus* to human corneocytes.

## Introduction

*Staphylococcus aureus* is a leading cause of bacterial infections in both healthcare settings and in the community, with high morbidity and mortality rates. This important pathogen can cause skin and soft tissue infections, which may become invasive resulting in difficult to treat infections like endocarditis, osteomyelitis, and pneumonia^1^. *S. aureus* is also a commensal bacterium, and it is estimated that the entire human population are asymptomatically colonised transiently during their lifetime with ~20% of individuals being permanently colonised^2^. Moreover, colonised individuals are at higher risk of infection and represent an important source of person-to-person transmission^3^.

Much of the prominence of *S. aureus* is due to its remarkable ability to adhere to and survive on the skin, its extensive arsenal of virulence factors, and an extraordinary ability to adapt rapidly to environmental fluctuations^1^. The first step for colonisation, and subsequent infection, is the adhesion of the bacteria to the skin. However, the bacterial factors that facilitate adhesion remain incompletely understood. Advances in understanding how *S. aureus* adheres to human skin are needed to inform new strategies for the elimination and prevention of *S. aureus* carriage in risk groups (e.g., elderly in nursing homes; premature babies; people who undergo dialysis or surgery; diabetic patients), which is in line with the World Health Organisation report establishing *S. aureus* as one of the high-priority multidrug-resistant organisms requiring research and development of new treatment and preventive approaches^4^.

The uppermost layer of the skin, the epidermis, acts as a physical barrier against invading microbes and allergens, being subdivided into stratified layers, each with a distinct composition of proteins. The stratum corneum is the outermost layer of the epidermis where flattened-anucleated keratinocytes, also known as corneocytes, are found. These are formed after the process of cell death known as cornification, and eventually, flake off the skin surface^5^. Replacing the plasma membrane in corneocytes is a structure known as the cornified envelope (CE). Loricrin, a 26 kDa protein, is the most abundant CE protein and presents a structure comprising three to four separate regions rich in glycine-serine residues predicted to form exposed omega loops^6^. Loricrin-deficient knockout mice were previously found to be more resistant to nasal colonisation by *S. aureus* than wild-type mice suggesting that this glycine-serine-rich CE protein is the main ligand recognised by *S. aureus*^7^.

**Fig. 1:**
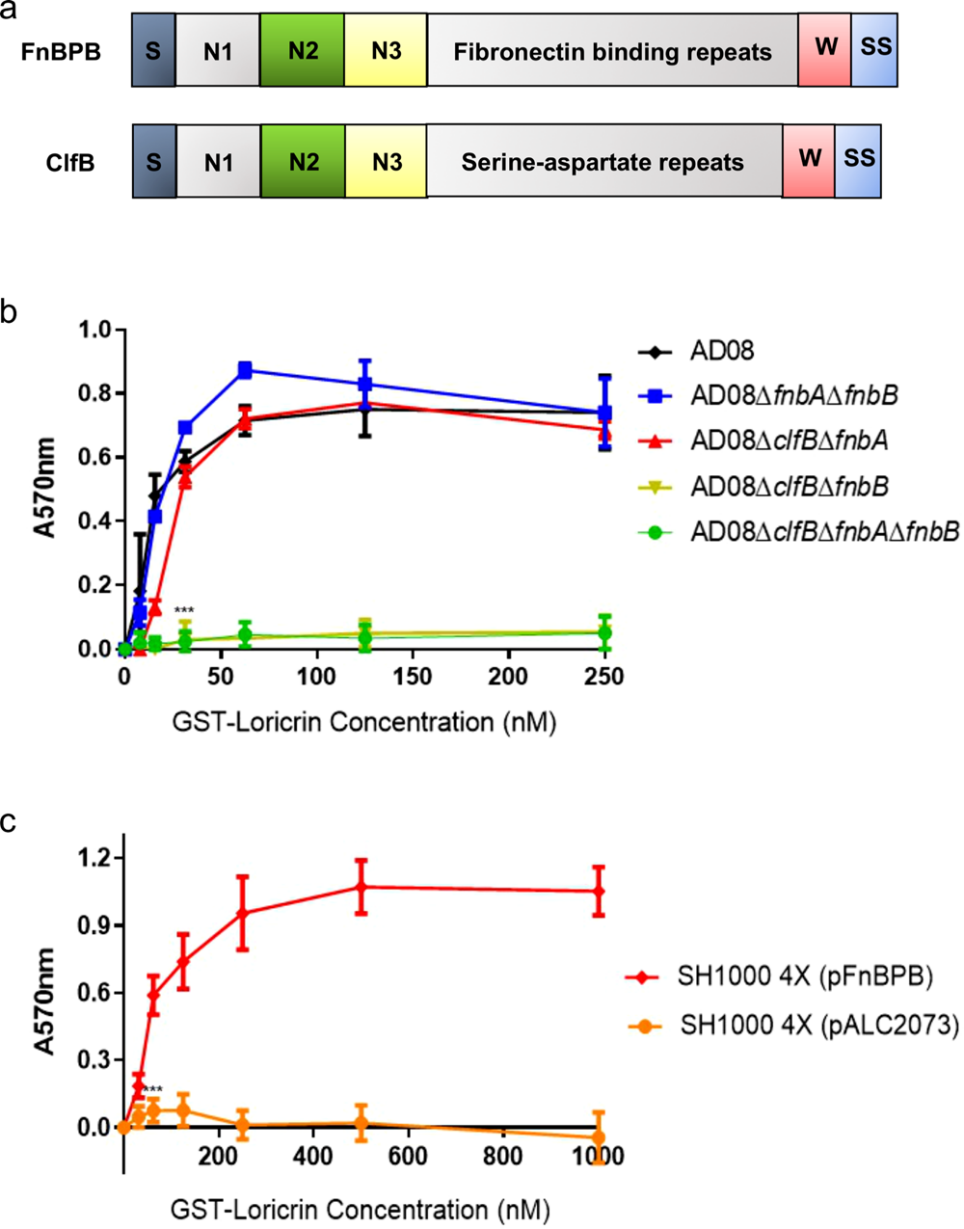
FnBPB and ClfB mediate the adherence of *S. aureus* to loricrin. **a** Schematic diagram showing the domain organisation of FnBPB and ClfB. The signal sequence (S) is followed by domains N1, N2, and N3. A series of fibronectin-binding repeats (FnBPB) or serine-aspartate repeats (ClfB) form a stalk that projects the N2 and N3 domains from the surface of the bacterium. The wall spanning region (W) is linked to the sorting signal (SS) which is processed by sortase A so that the C-terminus of the protein is anchored to peptidoglycan in the cell wall. **b** AD08 or mutants deficient in ClfB and/or FnBPA and/or FnBPB and, **c** *S. aureus* SH1000 deficient in ClfA, ClfB, FnBPA, and FnBPB [SH1000 4X] carrying pALC2073::*fnbB* (pFnBPB) or empty pALC2073 were grown to exponential phase (OD_600_ = 0.35), adjusted to an OD_600_ of 1.0 and incubated in microtiter plates coated with GST-tagged full length loricrin (GST-Loricrin) for 2 h. Following incubation, the wells were washed, adherent cells were stained with crystal violet, and the absorbance was read at 570 nm. The datum points on the graph represent the mean values of three independent biological experiments and error bars show the standard deviation. The ligand concentration given on the x-axis is the concentration of the solution used to coat the wells. **b** Statistical analysis was performed using a two-way ANOVA with a Dunnett´s multiple comparison test to compare variances between AD08 and the mutants at the 31.25 nM GST-loricrin concentration. ***P = 0.0001. **c** Statistical analysis was performed using a two-way ANOVA with Sidak´s multiple comparison test to compare variances between the strain carrying the plasmid expressing FnBPB and the empty plasmid at the 62.50 nM GST-loricrin concentration. ***P = 0.0000007. No symbol indicates *P* > 0.05.

Loricrin is a ligand for the *S. aureus* laboratory strain Newman, with adhesion mediated solely by the cell wall-anchored protein (CWAP) clumping factor B (ClfB). This interaction is critical for nasal colonisation^7^. ClfB is part of the microbial surface components recognising matrix molecules (MSCRAMM) family of cell wall-anchored proteins. ClfB and other MSCRAMMs such as clumping factor A (ClfA) and fibronectin-binding protein A and B (FnBPA and FnBPB, respectively) share a similar domain structure with an N-terminal A region comprising domains N1, N2 and N3, followed by a repeat region that connects the N3 domain to a sorting signal (Fig. 1a). The sorting signal is cleaved by sortase A and the MSCRAMM becomes covalently attached to the peptidoglycan in the cell wall^8^. Besides loricrin, ClfB binds to the alpha chain of fibrinogen, to the CE protein cytokeratin 10 (K10) and to corneodesmosin (CDSN), another glycine-serine-rich protein found in corneocytes^7,9–11^. The X-ray crystal structure of the ligand-binding region of ClfB shows that binding to ligands occurs by the “dock, lock and latch” (DLL) mechanism, forming an extremely stable bond equivalent to a covalent bond in terms of mechanical strength^12–16^. Steered molecular dynamics simulations in combination with AFM experiments showed that the extreme mechanical stability of various DLL complexes results from an intricate hydrogen bond network between ligand peptide and adhesin that distributes forces toward the peptide backbone^13^. DLL is facilitated by the N2 and N3 domains that act as the ligand-binding site. After peptide insertion into a binding trench located between N2 and N3 (“dock”), a C-terminal extension of the N3 subdomain undergoes a conformational change covering the peptide (“lock’), and beta-strand complementation in the N2 subdomain (“latch”), secures the peptide in place^7,12,17^. The binding of ClfB and FnBPB to CDSN mediates *S. aureus* adherence to corneocytes isolated from patients affected by atopic dermatitis (AD)^18–20,10^.

The aim of this study was to elucidate if FnBPB promotes adherence of *S. aureus* to loricrin and corneocytes from healthy human skin, and to gain an understanding of the strength and dynamics of the interaction. Ex vivo and in vitro experiments demonstrated that FnBPB mediates the adhesion of *S. aureus* to loricrin via very strong interactions, like those previously measured for the DLL mechanism and to human corneocytes. Isogenic mutants of *S. aureus* strain AD08 deficient in CWAPs allowed the importance of FnBPB in bacterial adherence to loricrin to be assessed. A *clfA, clfB, fnbA* and *fnbB* knockout mutant of *S. aureus* SH1000 complemented with a multicopy plasmid bearing the *fnbB* gene from AD08 (pFnBPB) encoding FnBPB, allowed further investigation. The strength of the interaction with loricrin and the binding site was investigated using atomic force microscopy (AFM)^21,22^, revealing very strong interactions similar to the ones observed by the DLL mechanism. The relevance of this interaction to the adherence of the bacteria to skin corneocytes was studied using healthy human cells. Strong binding forces in the same range as those measured between FnBPB and loricrin (ca. 2 nN) were detected between *S. aureus* expressing FnBPB and corneocytes. Blocking *S. aureus* adherence using recombinant N2N3 ClfB showed that both ClfB and FnBPB are key adhesins for *S. aureus* adherence to the skin, confirming that both proteins share the same corneocyte ligands. By combining in vitro, single-molecule, single-cell and ex vivo experiments, this study provides a deeper awareness of the repertoire of proteins involved in the adhesion processes, leading to a more precise comprehension of *S. aureus* colonisation.

## Results

### FnBPB promotes bacterial adherence to loricrin

We recently showed that FnBPB promotes adherence of *S. aureus* to the skin protein CDSN in patients affected by atopic dermatitis^10^. The FnBPB binding site within CDSN comprises the N-terminal glycine- and serine-rich region that is predicted to form a flexible glycine-serine loop. Loricrin also contains glycine-serine loops, raising the possibility that FnBPB might also recognise loricrin as a ligand. To investigate if FnBPB promotes adherence to loricrin, isogenic mutants of *S. aureus* strain AD08 deficient in ClfB and/or FnBPB and/or the related protein FnBPA were examined for their ability to adhere to purified GST-loricrin. The AD08 wild-type strain adhered to loricrin in a dose-dependent manner (Fig. 1b). However, neither AD08Δ*clfB*Δ*fnbB* nor AD08*∆clfB∆fnbA∆fnbB* adhered to loricrin, indicating that the presence of ClfB and/or FnBPB is necessary for adherence to loricrin and that FnBPA does not support adherence. Control experiments showing that AD08Δ*clfB*Δ*fnbB* is capable of adherence to fibronectin indicated that FnBPA is produced by this mutant (Supplementary Fig. 1) but fails to bind to loricrin. Interestingly, both AD08*∆fnbA∆fnbB* and AD08*∆clfB∆fnbA* adhered to loricrin in a manner that was not significantly different from the wild-type, indicating that expression of either ClfB or FnBPB is sufficient to support bacterial adherence (Fig. 1b). Next, a plasmid carrying the entire *fnbB* gene from AD08 was introduced into a mutant of *S. aureus* deficient in ClfA, ClfB, FnBPA and FnBPB (SH1000 4X) to generate the strain SH1000 4X (pFnBPB). SH1000 4X does not bind to loricrin (Supplementary Fig. 2) making it a suitable host for studying the interaction between FnBPB and loricrin in isolation. SH1000 4X (pFnBPB) adhered to immobilised recombinant GST-loricrin while the same mutant carrying an empty plasmid [SH1000 4X (pALC2073)] did not (Fig. 1c). Overall, these results indicate that FnBPB is capable of mediating adherence of *S. aureus* to loricrin.

### The FnBPB-loricrin bond is extremely strong

**Fig. 2:**
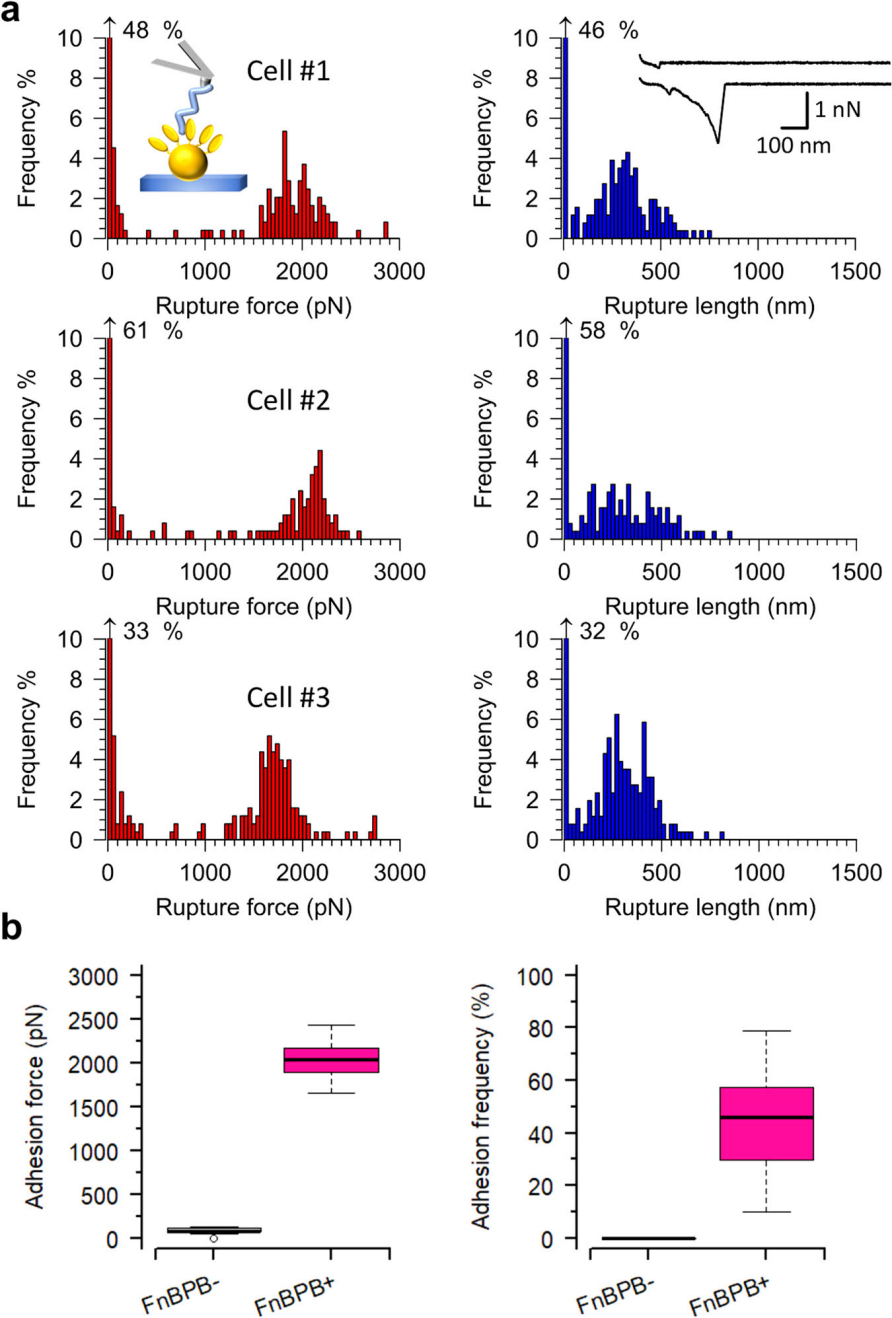
Strength of single interactions between FnBPB and loricrin. AFM tips modified with loricrin were used to probe single exponentially grown staphylococcal cells induced to express FnBPB. **a** Data for three representatives *S. aureus* SH1000 4X (pFnBPB) cells. Left. Histogram plots of rupture forces. Right. Histogram plots of rupture lengths with inset in the first cell showing representative curves. **b** Boxplots of adhesion forces and frequencies for *S. aureus* SH1000 4X (pALC2073) cells [FnBPB^−^] and *S. aureus* SH1000 4X (pFnBPB) cells [FnBPB^+^]. Whiskers show the range, the boxes the interquartile range, the thick lines within the boxes the median and open circles outliers. The differences in adhesion force (*P* = 1.3 × 10^−5^) and frequency (*P* = 1.6 × 10^−6^) between the FnBPB^−^ and FnBPB^+^ cells were tested using a two-sided Mann–Whitney *U*-test.

Individual FnBPB-loricrin interactions were probed using single-molecule force spectroscopy (SMFS). SH1000 4X (pALC2073) and SH1000 4X (pFnBPB) were probed in PBS with AFM tips grafted with loricrin. In the case of cells expressing FnBPB, two populations of rupture (also referred to as binding or adhesion) forces were observed. The first population had an average rupture force and rupture length of 122 ± 34 pN and 110 ± 50 nm (mean ± standard deviation [s.d.], Fig. 2a), respectively, and a binding frequency of 5 ± 3% (mean ± s.d., *n* = 220 adhesive curves from 12 cells, Fig. 2a). The second more abundant population had a much higher average binding force, rupture length and binding frequency of 2,079 ± 199 pN, 345 ± 87 nm and 46 ± 20% (mean ± s.d.), respectively (Fig. 2a). SH1000 4X (pALC2073) cells were also probed with loricrin tips (Fig. 2b and Supplementary Fig. 2). No large forces were observed for these cells, although weak bonds were detected. The rupture forces, lengths, and binding frequencies of these averaged at 84 ± 32 pN, 45 ± 34 nm and 10 ± 10%, respectively (mean ± s.d., *n* = 680 adhesive curves from 15 cells). In summary, these data show that the strong adhesion measured is due to FnBPB.

### Physical stress dramatically enhances the FnBPB-loricrin interaction strength

**Fig. 3:**
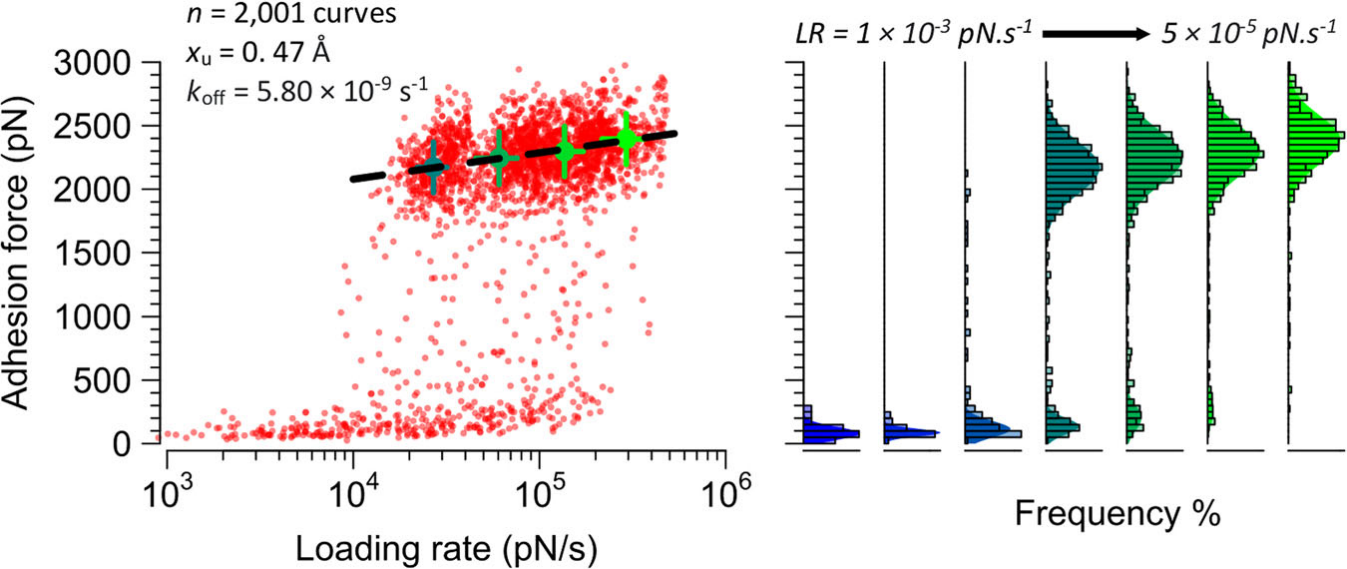
Mechanical stress activates the extremely strong FnBPB-loricrin interaction. Left: dynamic force spectroscopy plot of rupture force *vs* loading rate (*n* = 2305 curves from six cells). The dotted lines show the extrapolated Bell-Evans fit through the most probable (mean) rupture forces and loading rates for four log-equispaced loading rate bins shown as solid circles. Error bars are the standard deviations. Right: corresponding histograms and Gaussian fits of the data.

Bacteria need to establish firm adhesion to the skin or mucous surfaces to avoid being scraped off or washed away^23^. To achieve this, bacteria have evolved specific adhesion mechanisms, which are potentially activated by stress. To test this, the influence of physical tension on the FnBPB-loricrin interaction was investigated by measuring the rupture force vs loading rate (*LR*) using dynamic force spectroscopy (DFS) in which the tip retraction velocity is varied. Notably, bonds that ruptured under lower *LR* (1 × 10^3^ to 1.4 × 10^4^ pN s^−1^) were mainly weak (<500 pN, Fig. 3), while those that ruptured at higher *LR* (1.4 × 10^4^ to 5 × 10^5^ pN s^−1^) were predominantly very strong (>2 nN). This unusual behaviour points to activation of the FnBPB-loricrin interaction by mechanical stress, reminiscent of that described for DLL binding of ClfA^24^ and ClfB^15^. Fitting the high force-regime DFS data (i.e., between *LR* of 1.4 × 10^4^ and 5 × 10^5^ pN s^−1^) with the Bell-Evans model of forced dissociation kinetics revealed kinetic off rate (*k*_off_) and *x*_β_ (length of the energy barrier between the bound and unbound states) values of 5.8 × 10^−9^ s^−1^ and 0.47 Å, respectively. These results are within the range of previously reported MSCRAMM-ligand interactions^13,25^. The striking, unusual sharp force-enhanced adhesion is consistent with a catch bond^25,26^, i.e. a bond reinforced by tensile loading. Together with the previous result that FnBPB binds to loricrin with strong forces (2 nN), this raises the possibility that FnBPB utilises a DLL mechanism to bind loricrin.

### The strong FnBPB-loricrin bond most likely occurs through the DLL mechanism

**Fig. 4:**
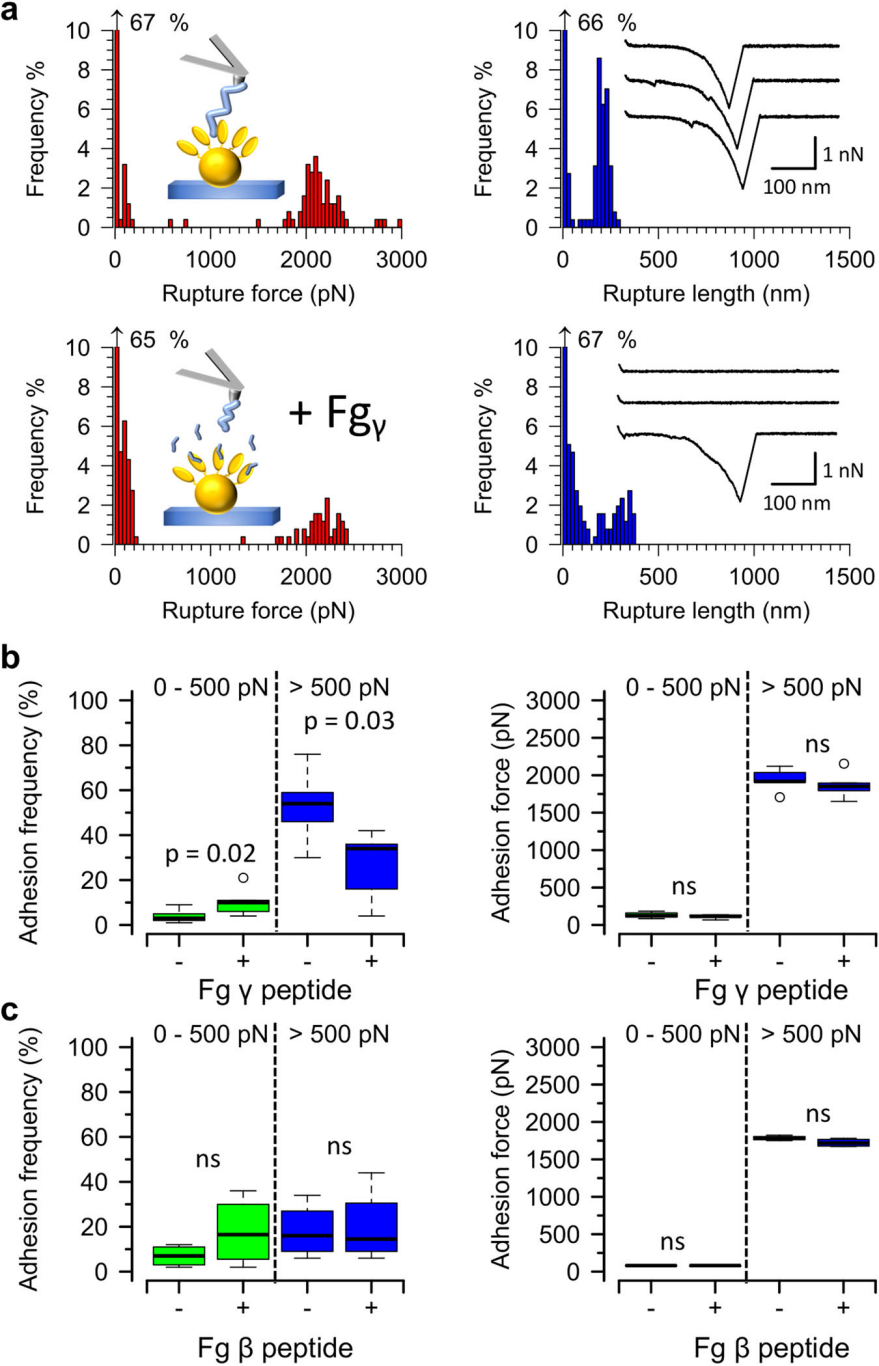
Inhibition of FnBPB-loricrin interaction by the fibrinogen gamma-chain peptide suggests a possible DLL mechanism. AFM tips modified with loricrin were used to probe single exponentially growing staphylococcal cells induced to express FnBPB. **a** Data for one representative *S. aureus* SH1000 4X (pFnBPB) cell before and after the fibrinogen (Fg) γ-chain C-terminal peptide (100 µg ml^−1^) was injected into the Petri dish. Left. Histogram plots of rupture forces. Right. Histogram plots of rupture lengths with inset showing three representative curves. **b** Boxplots of adhesion forces and frequencies for *S. aureus* SH1000 4X (pFnBPB) [FnBPB^+^] cells (*n* = 6 cells) before (−) and after (+) incubation with the Fg γ-chain C-terminal peptide. **c** Boxplots of adhesion forces and frequencies for SH1000 4X (pFnBPB) cells (*n* = 4 cells) before (−) and after (+) incubation with the Fg β-chain N-terminal peptide (Fg β peptide). Whiskers show the range, the boxes the interquartile range, the thick lines within the boxes the median and open circles outliers. Differences in data distributions were assessed using a two-sided Mann–Whitney *U*-test. ns not significant. *P* > 0.05.

FnBPB is believed to bind specifically to the extreme C-terminus of the fibrinogen gamma-chain via DLL, while such binding does not occur with the fibrinogen beta-chain^27–29^. To investigate if the DLL mechanism is involved in the interaction between FnBPB and loricrin, competitive blocking experiments were performed using short peptides. Cells of SH1000 4X (pFnBPB) were probed with loricrin-modified AFM tips before and after (Fig. 4a) injecting a peptide mimicking the fibrinogen gamma-chain C-terminal sequence (GEGQQHHLGGAKQAGDV). This did not appreciably alter the magnitudes of binding forces, neither in the high- nor in the low-force regime (Fig. 4b). However, treatment with the peptide decreased the frequencies of strong interactions, i.e. those exhibiting rupture forces superior to 500 pN, by ~twofold (53 ± 15%, mean ± s.d. for untreated vs 26 ± 16%, mean ± s.d. for treated cells, *n* = 6 cells, Fig. 4b). This was accompanied by an increase in the frequencies of weak interactions (in the range of 0 to 500 pN) from 4 ± 3% to 10 ± 6% (mean ± s.d.). No similar appreciable changes in frequencies of strong and weak interactions were observed when a peptide mimicking the fibrinogen beta-chain N-terminal sequence (NEEGFFSARGHRPLD) was used as a blocking agent (Fig. 4c). These results support our hypothesis that the DLL mechanism is involved in the very strong interaction between FnBPB and loricrin. However, the fact that incubation with the fibrinogen gamma-chain peptide did not also decrease the frequencies of the weaker interactions may point to the involvement of an additional binding site, which does not play a direct role in DLL binding.

### FnBPB promotes adherence of *S. aureus* to healthy corneocytes

**Fig. 5:**
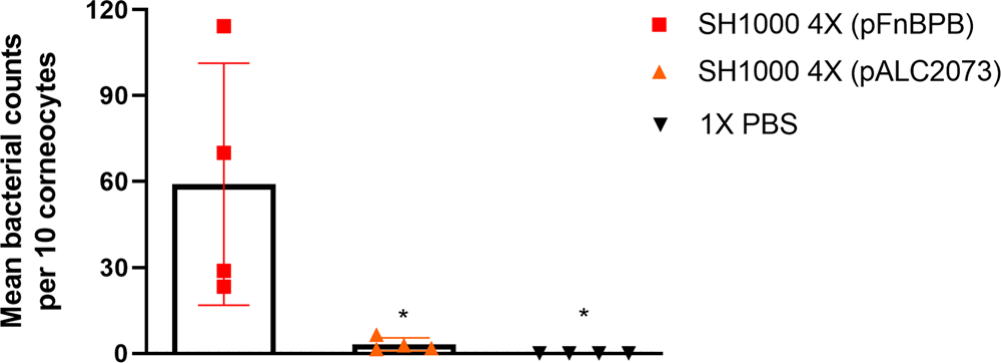
*S. aureus* adherence to healthy human corneocytes is mediated by FnBPB. SH1000 deficient in ClfA, ClfB, FnBPA, and FnBPB [SH1000 4X] carrying pALC2073::*fnbB* (pFnBPB) and pALC2073 were grown to exponential phase (OD_600_ = 0.35), incubated with healthy human corneocytes at 37 °C, washed, and adherent bacteria were stained with crystal violet. Corneocytes were visualised using light microscopy and the number of bacteria adhering to ten corneocytes was counted. Each datum point represents the mean number of bacteria per corneocyte. The bars represent the means of four independent experiments, with error bars showing the standard deviation. A no bacteria control was used where PBS alone was incubated with the corneocytes. Statistical analysis was performed using a one-way ANOVA with a Dunnett´s multiple comparison test to compare variances between *S. aureus* SH1000 4X (pFnBPB), *S. aureus* SH1000 4X (pALC2073), and the no bacteria control. *SH1000 4X (pFnBPB) *vs* SH1000 4X (pALC2073), *P* = 0.0186; SH1000 4X (pFnBPB) vs 1X PBS, *P* = 0.0139.

Loricrin is the most abundant human CE protein and the interaction between ClfB and loricrin is proposed to facilitate bacterial adherence to desquamated nasal epithelial cells^7^. Here we sought to determine if FnBPB can promote adherence to human skin corneocytes. *S. aureus* SH1000 4X (pFnBPB) and *S. aureus* SH1000 4X (pALC2073) were incubated on corneocytes sampled from the stratum corneum of healthy subjects. *S. aureus* SH1000 4X (pFnBPB) bound to corneocytes at a significantly higher level than *S. aureus* SH1000 4X (pALC2073), indicating that FnBPB contributes significantly to corneocyte adhesion (Fig. 5).

### FnBPB and ClfB share the same ligands to promote *S. aureus* adherence to corneocytes

**Fig. 6:**
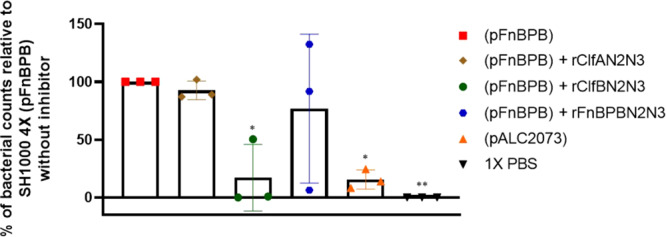
Recombinant ClfB N2N3 reduces *S. aureus* FnBPB binding to healthy human skin corneocytes. SH1000 4X (pFnBPB) and SH1000 (pALC2073) were grown in TSB to exponential phase (OD_600_ = 0.35), adjusted to an OD_600_ = 1.0 and incubated with tape strips containing corneocytes previously treated with rClfAN2N3 or rClfBN2N3 or rFnBPBN2N3. Each datum point is from a different biological replicate, and the bars represent the means of three independent experiments, with error bars showing the standard deviation. A no bacteria control was used where PBS alone was incubated with the corneocytes. Tape strips were washed, and adherent bacteria were stained with crystal violet. The number of bacterial cells adhering to ten corneocytes was counted and the percentage of adherence is shown in comparison with the total adherence of SH1000 4X (pFnBPB) to corneocytes that were not pretreated with recombinant protein. Statistical analysis was performed using a one-way ANOVA with a Dunnett´s multiple comparison test to compare variances between *S. aureus* SH1000 4X (pFnBPB) adhering to corneocytes without recombinant protein to the *S. aureus* SH1000 4X (pALC2073) adhering to corneocytes without recombinant protein, SH1000 4X (pFnBPB) adhering to corneocytes with recombinant protein, and the no bacteria control.*P = 0.0160 (SH1000 4X (pFnBPB) vs SH1000 4X (pALC2073) and *P* = 0.0178 (SH1000 4X (pFnBPB) vs SH1000 4X (pFnBPB) + rClfBN2N3), *P* = 0.0051 (SH1000 4X (pFnBPB) vs 1X PBS).

To investigate whether loricrin and possibly other ClfB ligands, were also the target ligand of FnBPB in healthy corneocytes, we used recombinant N2N3 subdomains (rClfBN2N3 or rFnBPBN2N3) to block the corneocyte ligands before adding the bacterial suspension to the skin cells. rClfAN2N3 was also used, as a control, as it is known that ClfA does not promote *S. aureus* adherence to corneocytes^30,31^. On corneocytes where rClfBN2N3 was added, the adherence of SH1000 4X (pFnBPB) was significantly reduced (Fig. 6), suggesting that FnBPB promotes *S. aureus* adherence to healthy human corneocytes by interaction with the same ligand(s) that ClfB binds to. The addition of rClfAN2N3 did not affect *S. aureus* adherence to corneocytes. Similarly, no reduction in overall adherence occurred when corneocytes were treated with rFnBPBN2N3, but unlike with the control protein (rClfAN2N3) *S. aureus* aggregates were observed adhering to the corneocytes. No differences were observed in adherence of *S. aureus* SH1000 4X (pALC2073) to corneocytes pretreated with recombinant proteins (Supplementary Fig. 3). Together these results indicate that FnBPB mediates *S. aureus* adherence to corneocytes and that ClfB and FnBPB share the same ligand(s).

### FnBPB promotes strong adhesion of *S. aureus* to human corneocytes

**Fig. 7:**
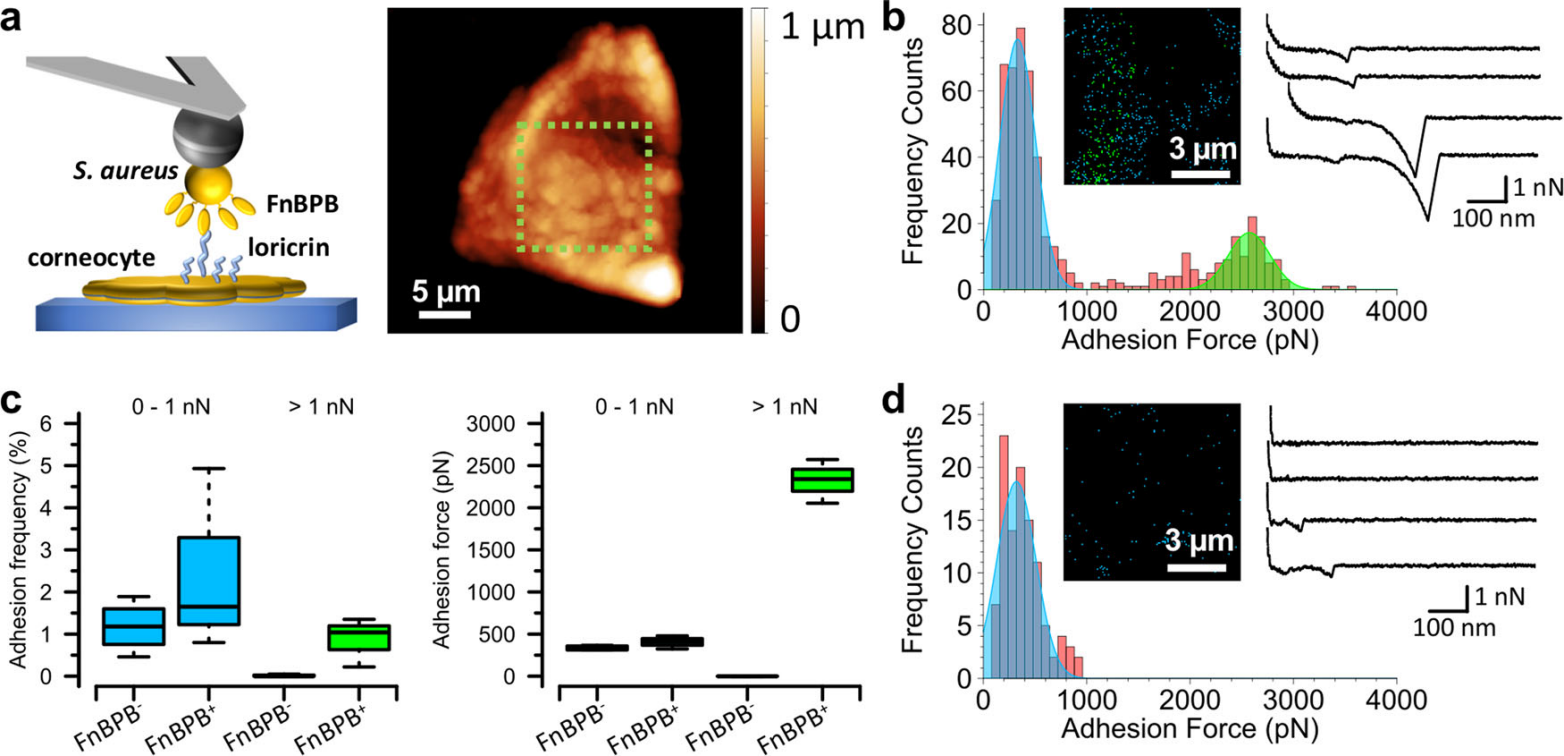
FnBPB expressed on living *S. aureus* cells forms ultrastrong single molecular complexes with ligands exposed on corneocyte surfaces. Binding forces between *S. aureus* and corneocytes. **a** Single bacterial cells were attached to a dopamine-treated (wet adhesive) colloidal probe and used to force probe corneocytes on tape strips in quantitative imaging (QI) mode. Height image of a single corneocyte produced with an *S. aureus* probe. The green zone indicates where subsequent QI adhesion data were recorded. **b** Representative data for *S. aureus* SH1000 4X (pFnBPB) cells. Left, histograms of binding forces with inset of the corresponding adhesion map. Black pixels show where zero adhesion was measured and blue pixels show where adhesive interactions between 0 and 1000 pN were detected. Green pixels are where adhesive interactions greater than 1000 pN were detected. Right, representative force-extension curves. **c** Boxplots of adhesion forces and frequencies for *S. aureus* SH1000 4X (pALC2073) cells [FnBPB^−^] and *S. aureus* SH1000 4X (pFnBPB) cells [FnBPB^+^] used to probe corneocytes. Whiskers show the range, the boxes the interquartile range, the thick lines within the boxes the median and open circles outliers. *n* = 772 adhesive curves from three independent bacterial cells. Adhesion frequency values for FnBPB^−^ and FnBPB^+^ cells were subjected to statistical testing using a two-sided Wilcoxon rank-sum test with continuity correction. For adhesion forces >1 nN, *P* = 0.0436 and for adhesion forces 0–1 nN, *P* = 0.6286. **d** Representative data for the control *S. aureus* SH1000 4X (pALC2073) strain that does not express FnBPB. *n* = 1209 adhesive curves from three independent bacterial cells.

To test the relevance of ultrastrong binding between FnBPB and loricrin for adhesive interactions between *S. aureus* and corneocytes, we used single-cell force spectroscopy (SCFS) to directly probe interactions between single living *S. aureus* cells and corneocytes obtained from the stratum corneum of healthy subjects. A colloidal probe bound to a single living *S. aureus* bacterium was used to force probe a corneocyte using the quantitative imaging mode (Fig. 7a). A rapid approach and retract speed of 30 µm s^−1^ allowed resolution of the surface relief of the cells and high-resolution localisation of detected binding events (Fig. 7a). Using this approach for SH1000 4X (pFnBPB) delivered well-resolved force-distance curves in which adhesive peaks exhibiting typical polymer extension were often seen (Fig. 7b). Two populations were observed for the adhesion forces: (i) a population rupturing at a weaker force of 405 ± 78 pN (mean ± s.d., *n* = 1209 adhesive curves from three bacterium-corneocyte pairs) and (ii) a second population that ruptured at a high force of 2322 ± 260 pN (*n* = 1209 adhesive curves from three bacterium-corneocyte pairs), (Fig. 7a, c and Supplementary Fig. 4a) representing ~26% of all adhesive events. The adhesion images suggest a heterogeneous distribution of the ligand (Fig. 7a and Supplementary Fig. 4a). The near absence of serial adhesive peaks, intermediate adhesion force populations or adhesion events of >4000 pN attest to both the weaker and stronger binding populations representing single molecular complexes. While the weaker binding population was still present in the FnBPB- strain (335 ± 27 pN, *n* = 772 adhesive curves from three bacterium-corneocyte pairs, Fig. 7c and Supplementary Fig. 4), the stronger binding population was entirely absent (Fig. 7d and Supplementary Fig. 4), conclusively proving the involvement of FnBPB in the strong-binding population. Thus, similar strong binding forces were measured for bacteria expressing FnBPB binding to loricrin and binding to corneocytes suggesting that the bindings partners interact in situ, on the surface of the bacterium and the surface of the corneocyte.

## Discussion

*S. aureus* is a commensal of humans, with colonisation being a significant risk factor for subsequent infection. Targeted decolonisation with topical antibiotics is used to prevent infection in specific groups but the efficacy of this approach is threatened by antibiotic resistance^1^. Advances in understanding the bacterial and host factors that act during the very first steps of the establishment of colonisation are urgently needed. Here, we identify loricrin as a ligand for an *S. aureus* cell wall-anchored protein FnBPB and provide a comprehensive analysis of the interaction. Using bacteria expressing FnBPB, we show that the binding is very strong using SMFS and DFS. Then we demonstrate that blocking FnBPB with a peptide that binds to the ligand-binding trench located between domains N2 and N3, reduces the frequencies of strong FnBPB-loricrin interactions. We also show that FnBPB facilitates the adherence of *S. aureus* to corneocytes using an ex vivo adhesion model with corneocytes from healthy donors and that blocking the corneocytes with recombinant ClfB N2N3 domains reduces the interaction of bacteria expressing FnBPB with corneocytes, indicating that ClfB and FnBPB share the same corneocyte ligand(s).

Our previous work suggested that the cell wall-anchored protein ClfB was the sole *S. aureus* adhesin promoting adhesion to loricrin^7^. However, Mulcahy et al.^7^ studied the laboratory strain Newman, which produces a truncated FnBPB protein that is not cell wall anchored^32^. Here using a clinical isolate with functional FnBPB (and FnBPA) proteins we demonstrate that FnBPB is a second loricrin-binding protein of *S. aureus* and can support adhesion to loricrin independently of ClfB. Despite FnBPA and FnBPB sharing 48% amino acid identity in their N2N3 subdomains, FnBPA does not mediate bacterial adherence to loricrin. This mirrors what we have previously found with CDSN where FnBPB and ClfB, but not FnBPA, facilitate bacterial adherence. Both loricrin and CDSN contain glycine- and serine-rich sequences that are predicted to form flexible loop structures^33,34^. ClfB has binding sites throughout the glycine-serine rich loop regions of loricrin^7^. Both ClfB and FnBPB bind to a glycine-serine rich region at the N-terminus of CDSN^10^. We hypothesise that FnBPB may also recognise glycine-serine loops in loricrin explaining how ClfB and FnBPB share these ligands and why binding of recombinant ClfB inhibits FnBPB-mediated adherence. The relative contribution of ClfB and FnBPB to corneocyte adherence is likely to vary depending on the *S. aureus* strain and the level of each protein being expressed. In addition, the cell wall-anchored protein SasG is expressed by some strains of *S. aureus* and mediates adherence to corneocytes, but not by binding to loricrin^35^. Thus, further studies are needed to understand the full complement of proteins contributing to corneocyte adherence and their relative contributions.

Several studies have reported that DLL interactions between MSCRAMMs and their ligands are extremely strong (force of c. 2 nN)^13,24,25,28^. Here we probed individual FnBPB-loricrin interactions using SMFS and measured a high binding force (2 nN). The binding of ClfB to loricrin was reported to be remarkably strong with a strength similar to that measured for the FnBPB-loricrin interaction in the present study^15^. Most polypeptide folds generally unfold at forces well below 500 pN^36–38^. However, because of the interaction geometry, the A domain of FnBPB bound to loricrin is unlikely to unfold at 2 nN^13^. The length of fully extended GST-loricrin used here is (0.35 nm × 538 amino acids) c. 190 nm, and the length of the peptide sequence between the FnBPB A region and the anchorage point at the cell wall in the C-terminal (0.35 nm × 424 amino acids) is c. 150 nm. The sum of these values and the size of the folded A region (< 10 nm) equates to ~350 nm, which agrees with the mean rupture length measured in our study. Such extreme forces structurally originate from the DLL mechanism (or variations of it), which involves locking of the docked target peptide sequence by a C-terminal segment of the adhesin followed by latching of this complex onto a neighbouring domain in the adhesin^8,14^. Steered molecular dynamics simulations in combination with AFM experiments showed that the extreme mechanical stability of various DLL complexes results from an intricate hydrogen bond network between the ligand peptide backbone and the adhesin^13^.

The crystal structure of the N2N3 subdomains of FnBPB has not yet been solved, but structures of related MSCRAMMs provide information about the ligand-binding mechanism. ClfB is predicted to bind to loricrin using the DLL mechanism since introducing amino acid substitutions into the trench located between domains N2 and N3 eliminates loricrin binding^7^. Similarly, the substitution of key residues within the ligand-binding trench of FnBPB eliminates binding to fibrinogen^39^. Here we show that a peptide mimicking the fibrinogen gamma-chain sequence inhibits the interaction between FnBPB and loricrin, suggesting that fibrinogen and loricrin share a binding site within FnBPB. The interaction between FnBPB and loricrin may be more extensive than the interaction with the fibrinogen gamma-chain as the peptide blocked only very strong interactions indicating that weaker interactions may occur outside of the ligand-binding trench and involve other residues. In the case of the ClfA-fibrinogen interaction, two binding sites have been implicated: (i) a high-affinity binding site located on top of the N3 subdomain that, counterintuitively, appears to support mechanically weaker binding and does not involve the fibrinogen gamma-chain C-terminal sequence and (ii) a lower affinity binding site located between the N2 and N3 domains that bind the fibrinogen gamma-chain C-terminal sequence and support a very stable interaction between ClfA and fibrinogen^24,40^. We are currently working to solve the crystal structure of FnBPB in complex with loricrin to gain a better understanding of this interaction. This combined with a comprehensive biochemical analysis of the interaction will provide further insight into the mechanism of binding by FnBPB.

Bacteria are subject to high physical stresses in vivo, a phenomenon of major importance for function. It is known that shear stress has an important role in strengthening bacteria–host interactions^26,41^. More specifically *S. aureus* encounters mechanical stresses such as fluid flow, washing, and scraping on the surface of the stratum corneum, necessitating strong attachment during colonisation^23^. Here we studied the influence of physical tension on the FnBPB-loricrin interaction using DFS. The remarkable, sharp force-enhanced adhesion observed points to activation of the FnBPB-loricrin interaction by mechanical stress, reminiscent of that described for DLL binding of ClfA^24^ and ClfB^15^. Moreover, the striking unusual behaviour is consistent with a catch bond (i.e., a bond reinforced by tensile loading)^25,26^, and maybe a mechanism used by FnBPB to strengthen attachment under shear stress conditions, as encountered on the skin^23^.

Treating normal corneocytes with recombinant ClfB N2N3 domains reduced FnBPB-mediated adherence of *S. aureus*, while treatment with rClfAN2N3 did not. This is consistent with FnBPB and ClfB sharing a ligand on corneocytes while ClfA, a protein implicated in infection but not colonisation^42–44^, does not. Surprisingly, treatment with rFnBPBN2N3 did not affect the adhesion of *S. aureus*. We observed aggregation of bacteria when added to corneocytes preincubated with rFnBPB. We speculate that this aggregation is due to the ability of the N2N3 subdomains of FnBPB to undergo homophilic interactions, something we have shown previously for FnBPA^45,46^. In this case, rather than inhibiting interactions with corneocyte ligands, bound rFnBPBN2N3 acts as a bridge between the corneocyte and bacteria expressing FnBPB. Nevertheless, our data indicate that ClfB and FnBPB share a ligand on the surface of corneocytes. Previously we found that FnBPB and ClfB promote *S. aureus* adhesion to corneocytes in AD patients through interaction with CDSN^10^. CDSN is displayed aberrantly on the tips of villus-like projections that are abundant on the surface of corneocytes from patients with severe AD due to low natural moisturising factor (NMF) levels but confined to the periphery of healthy corneocytes where NMF is high^47^. Since our study involved corneocytes from normal healthy donors with no history of AD it is unlikely that NMF levels are low or that CDSN is accessible to bacteria. We did not attempt to block the interaction between *S. aureus* and corneocytes using soluble loricrin since loricrin, fibrinogen (and possibly CDSN) bind to a common site in FnBPB and therefore this experiment would only tell us that the ligand-binding trench of FnBPB was being blocked, not that the interaction with loricrin was inhibited specifically. We know that loricrin is abundant in the CE and that it is bound by *S. aureus* on the surface of desquamated nasal epithelial cells. We, therefore, believe that loricrin is the main target for both adhesins to adhere to normal skin. This is supported by SCFS experiments showing that the strength of binding of *S. aureus* expressing FnBPB to corneocytes is similar to the strength of the FnBPB-loricrin interaction. In addition, rupture lengths correspond with those measured in SMFS experiments with loricrin tips, further supporting the notion the ligand on corneocytes bound strongly by FnBPB is loricrin. We predict that FnBPB also promotes bacterial adhesion to desquamated nasal epithelial cells through an interaction with loricrin potentially contributing to nasal colonisation. While loricrin production is usually confined to the upper layers of the epidermis, it can be present at sites of tissue damage^48^ where it would provide a ligand for FnBPB. In support of this, we have recently shown that loricrin is recruited to the outer walls of subcutaneous skin abscesses formed during *S. aureus* infection in mice^49^.

In summary, this study elucidates a key interaction involved in the adhesion of *S. aureus* to skin. We show that FnBPB, a protein previously implicated in invasive infection^50–56^, also promotes adhesion to corneocytes, facilitating colonisation of the stratum corneum. The likely importance of FnBPB in colonisation was not appreciated previously due to the failure of the widely-used laboratory strain Newman to produce functional FnBPB. Our study provides important insights that aid in our understanding of the host-bacterium interactions that occur during colonisation. This knowledge will benefit efforts to develop strategies to prevent or eliminate colonisation. For example, we propose that specifically targeting bacterial adhesins such as FnBPB will reduce adhesion to corneocytes thus interfering with *S. aureus* colonisation.

## Methods

### Bacterial strains and plasmids

All strains are listed in Supplementary Table 1. *S. aureus* strains were grown in tryptic soy broth (TSB) at 37 °C with shaking (200 rpm). *E. coli* strains were grown at 37 °C in lysogeny (L) broth with shaking (200 rpm). Antibiotics were added to the medium where appropriate: ampicillin (100 μgml^−1^), chloramphenicol (10 μg ml^−1^), and anhydrotetracycline (0.3 μg ml^−1^).

All plasmids are listed in Supplementary Table 2. Plasmids pALC2073 and pFnBPB were isolated from *E. coli* IM08B and used to transform electrocompetent SH1000 4X according to the method described by Löfblom et al.^57^. Plasmid DNA (5 µg) was added to the cells, transferred to a 1 mm electroporation cuvette (Bio-Rad) and pulsed at 21 kV/cm, 100 Ω and 25 µF. About 1 ml of BHI medium supplemented with 500 mM sucrose was added and the cells were incubated at 37 °C for 1 h before plating on BHI agar containing chloramphenicol and incubated at 37 °C overnight.

### Recombinant protein expression and purification

Cultures of *E. coli* Topp3 or XL-1 Blue carrying expression vectors were grown to an optical density at 600 nm (OD_600_) of between 0.6 and 1. Expression was induced by isopropylß-d-1- thiogalactopyranoside (1 mM; VWR Chemicals) for 3 to 5 h, after which the bacteria were harvested and lysed using a French Pressure Cell at 1500 psi. GST and recombinant GST-tagged human loricrin (GST-loricrin), were purified by glutathione affinity chromatography on a GSTrap FF purification column (GE Healthcare) according to the manufacturer’s instructions^7^. Recombinant N2N3 subdomains of ClfA (residues 221–559, rClfAN2N3)^58^, ClfB (residues 201–542, rClfBN2N3)^7^, and FnBPB (residues 162–480, rFnBPBN2N3)^27^ were expressed with an N-terminal hexahistidine tag and purified using Ni^2+^ affinity chromatography. The purified proteins were analysed by SDS-PAGE. Protein concentrations were determined using a BCA Protein Assay Kit (Pierce) as per the manufacturer’s instructions.

### SDS-PAGE

Purified recombinant His-tagged N2N3 proteins (6 μM) were boiled for 10 min in Laemmli final sample buffer (Sigma) and separated on polyacrylamide gels. The gels were stained using InstantBlue (Expedeon).

### Bacterial adherence assay

The wells of a microtiter plate (Nunc Maxisorb) were coated with recombinant GST-loricrin diluted in sodium carbonate buffer (0.1 M NaHCO_3_, pH 9.6) and incubated overnight at 4 °C. The plates were washed three times with PBS and wells were blocked with 5% (wt/vol) bovine serum albumin (Fisher) for 2 h at 37 °C. Bacterial cultures were grown to exponential phase (OD_600_ = 0.35) in TSB. Strains harbouring plasmid pALC2073 or its derivative were grown in TSB containing chloramphenicol and expression was induced by adding anhydrotetracycline once the OD_600_ reached 0.18 and allowing the bacteria to grow to an OD_600_ = 0.35. Bacteria were washed and adjusted to an OD_600_ = 1.0 in phosphate-buffered saline (PBS) and 100 μL of bacterial suspension was added to each well of the microtitre plate. The plate was incubated for 2 h at 37 °C, and unbound cells were washed off the plate with PBS before adding formaldehyde (25% vol/vol; Sigma) to fix adherent cells. Adherent bacteria were stained using crystal violet (0.5% wt/vol), washed with PBS, and solubilised with acetic acid (5% v/v). Absorbance was measured at 570 nm in an ELISA plate reader.

### Atomic force microscopy (AFM)

#### Single-molecule force spectroscopy (SMFS)

Lyophilised GST-loricrin (kept at 4 °C) was equilibrated to room temperature and dissolved in PBS at 0.1 mg ml^−1^. Gold-coated PNP-Tr-Au AFM tips (NanoWorld) were immersed for 16 h in an ethanolic solution containing 16-mercaptododecahexanoic acid (0.1 mM) and 1-mercapto-1-undecanol (0.9 mM). The tips were then washed with ethanol, dried under a stream of N_2_ and immersed for 30 min in an aqueous solution of *N*-hydroxysuccinimide (NHS, 10 mg ml^−1^) and 1-ethyl-3-(3- dimethylaminopropyl)-carbodiimide (EDC, 25 mg/mL). The resulting NHS-carboxyl ester exposing tips were rinsed with ultrapure water and submerged in a loricrin (0.1 mg ml^−1^) solution for 1 h. Finally, the tips were gently rinsed with PBS and stored in PBS until they were used for experimentation. Prior to measurements, AFM tip spring constants were determined empirically by the thermal noise method^59^. Stationary phase (overnight) cultures of bacteria were collected by centrifugation, washed once with TSB, diluted 100× in the same medium and cultured with shaking at 37 °C to an OD_600_ = 0.18, at which point anhydrotetracycline was added (300 ng ml^−1^) and the bacteria further cultured until an OD_600_ = 0.35 was reached. Then the bacteria were again collected by centrifugation, washed twice with PBS, and resuspended in a volume of PBS equal to the original culture volume. A small volume of cellular suspension was then deposited on the surface of a polystyrene Petri dish and the cells were allowed to adhere for 20 min. Finally, the cells were washed, the Petri dish filled with PBS and AFM experimentation was performed. AFM was done using a JPK NanoWizard^®^ 4 NanoScience AFM and JPK NanoWizard^®^ Control Software Version 6.1.115. Force-distance curves were collected in force mapping mode using a constant approach and retraction speed of 1 µm s^−1^, a ramp length of 1 µm, a contact force setpoint of 150 pN, zero dwell time, and a closed *z*-loop. A force map was collected on an area measuring 500 × 500 nm on top of a single bacterium. In DFS experiments all the parameters described above were the same except for the retraction speed, for which the following range was used: 1, 2.5, 5 and 10 µm s^−1^. For all force-displacement curves, the last rupture peak was fit using the extensible worm-like chain (eWLC) model of polymer extension^60^. Rupture forces and lengths, as well as *LR*, were obtained along with eWLC fit parameters with the JPK data processing software (version 6.1.125), which uses an algorithm to calculate these values from the fit data.

#### Inhibition of interaction using short peptides

After performing the first recording of a force map on top of a single bacterium using a loricrin grafted probe as indicated above, the fibrinogen gamma-chain C-terminal or beta-chain N-terminal peptides (Genscript) freshly dissolved in PBS (2 mg/ml) were injected into the Petri dish to a final concentration of 0.1 mg ml^−1^ and after 15 min another force map was recorded on the same bacterium. The Petri dish was changed and the whole procedure was repeated for each cell.

#### Single-cell force spectroscopy to probe interactions between single living S. aureus cells and corneocytes

Single-cell probes were prepared as previously described and SCFS experiments were performed^19,61^. Briefly, tipless NPO-10 cantilevers (Bruker) to which single silica microspheres (6.1 µm diameter) were glued to the end were placed for 1 h in a polydopamine solution. The spring constant of such a colloidal probe was first determined empirically using the thermal noise method (typically ~0.06 N m^−1^). Then it was brought into contact with a single *S. aureus* cell weakly adhered to the surface of a treated polystyrene dish in PBS (localised using an inverted optical microscope) for 1 min allowing the bacterium to bind to it. Next, the probe was transferred to an area in the same dish where a piece of corneocyte-exposing tape strip was previously pasted using double-sided tape. The transparent tape strips allowed observation of the corneocytes with the inverted microscope. Force measurements were subsequently performed initially in the quantitative imaging mode, using the following parameters: a contact setpoint of 1 nN, a ramp length of 1 µm, a constant approach and retraction speed of 30 µm s^−1^ and a scan area of 15 µm × 15 µm (128 × 128 pixels) on top of a corneocyte. Subsequently, a conventional force volume map was recorded on a smaller area (2 µm × 2 µm, 16 × 16 pixels) where adhesive events were observed in the quantitative imaging mode adhesion image using similar force spectroscopy parameters apart from a slower constant approach and retraction speed of 1 µm s^−1^.

### Corneocyte collection

Corneocytes were collected from the volar part of an adult volunteer’s forearm using a tape stripping method^62^. A circular adhesive tape strip (3.8 cm^2^; D-Squame, Monaderm) was attached to the forearm, and a standardised force was applied for 10 s. The tape strip was gently removed with tweezers and a second strip was attached to the exact same site. The process was repeated until eight consecutive tape strips were sampled from the same site and the seventh and eighth consecutive strips were used to measure bacterial binding. The tape strips were used immediately after collection or stored frozen at −80 °C until use.

### Corneocyte adherence assay

A single tape strip from a healthy volunteer was cut in half, and each half was placed into a different well of a six-well plate. Bacteria were grown to an exponential phase as described for the adherence assay. Bacteria were washed and adjusted to an OD_600_ = 1.0 in PBS. 300 μL of the bacterial suspension, or PBS as a no bacteria control, was placed onto each half of the tape strip and incubated for 1 h at 37 °C. Following incubation, 2 mL of PBS was added to each well to avoid further adhesion, and each half strip was washed three times with 1 ml PBS. The tape strips were allowed to dry for 30 min. The cells were fixed using the cytological fixative CytofixxTM (Cellpath) and allowed to dry for a further 30 min. Each half strip was stained using crystal violet (0.0025%; wt/vol), washed in PBS, and allowed to dry for 30 min. Strips were mounted onto glass slides, and coverslips (25 × 50 mm) were overlaid using the D.P.X. mountant (BDH). Corneocytes were visualised under a microscope at 1000X magnification, and the number of adherent bacteria per corneocyte was counted (single-blind).

### Inhibition of *S. aureus* adherence to corneocytes using recombinant protein

A single tape strip from a healthy volunteer was cut in four, and each quadrant was placed into a different well of a six-well plate. Volumes of 100 μL of rClfAN2N3, rClfBN2N3 or rFnBPBN2N3 (all at a concentration of 6 μM) were placed onto each quadrant of tape strip and incubated at room temperature for 40 min. After incubation, each quadrant was washed with 1 mL of PBS and left to dry for 5 min. Bacteria were grown as described above and adjusted to OD_600_ = 1.0. The suspension of bacteria or PBS (100 μL) was placed onto each quadrant and incubated for 1 h at 37 °C. Following incubation, 1 mL of PBS was added to each well, and each quadrant was washed with 1 mL of PBS. Adherent cells were fixed, stained and the quadrants were mounted onto glass slides with coverslips as described above. Corneocytes were visualised under a microscope at 1000X magnification, and the number of adherent bacteria per corneocyte was counted (single-blind).

### Ethical approval

Our research complies with all relevant ethical regulations. Ethical approval for the collection and use of human corneocytes from adult volunteers was obtained from the Trinity College Dublin Faculty of Health Sciences Ethics Committee, Ireland (ref. 2020402). Personal information such as sex and age was not collected. Written informed consent was obtained from the participants and all participants were over the age of 18. No compensation was provided to the participants.

### Statistics and reproducibility

For the bacterial adherence assay, statistical analysis was performed using the last datum point before binding saturation was reached. Statistical analyses were performed using GraphPad Prism version 8.0.2. A minimum of three biological replicates (three independent experiments) were conducted for each experiment. For experiments with corneocytes, cells from different donors were used for each of the biological replicates.

For SMFS and SCFS experiments, statistical analyses were performed and graphs were drawn with the R programming language. For DFS data, the Bell-Evans model was fit through the means of rupture force vs *LR* calculated for log-equispaced *LR* bins using nonlinear least-squares regression and the Port algorithm in RStudio version 1.3.1073. Sample sizes and replicates are reported in the figure legends. Experiments were repeated at least twice. Differences in data distributions between groups were analyzed using two-way Mann–Whitney U tests. For all experiments, a *P* value <0.05 was considered significant.

### Reporting summary

Further information on research design is available in the Nature Research Reporting Summary linked to this article.

## Supplementary information

Supplementary Information

Reporting Summary

## Data Availability

All data generated or analysed during this study are included in the published article (and its supplementary information files).  Source data are provided with this paper.

## Source data

Source Data
