# Supplementary material for: Fibronectin binding protein B binds to loricrin and promotes corneocyte adhesion by Staphylococcus aureus
Source: Nat Commun. 2022 May 6;13:2517. doi: 10.1038/s41467-022-30271-1 (PMC9076634; doi:10.1038/s41467-022-30271-1)
Supplement: Supplementary file 1 — Supplementary Information [file 41467_2022_30271_MOESM1_ESM.pdf]

SUPPLEMENTARY INFORMATION

Fibronectin binding protein B binds to loricrin and promotes corneocyte adhesion by *Staphylococcus aureus*

Thaina M. da Costa[1§], Albertus Viljoen[2§], Aisling M. Towell[1], Yves F. Dufrêne[2,3*], Joan A. Geoghegan[1,4*]

[1]Department of Microbiology, Moyne Institute of Preventive Medicine, School of Genetics and Microbiology, Trinity College Dublin, Dublin 2, Ireland.
[2]Louvain Institute of Biomolecular Science and Technology, UC Louvain, Croix du Sud, 4-5, bte L7.07.07, B-1348 Louvain-la-Neuve, Belgium.
[3]Walloon Excellence in Life sciences and Biotechnology (WELBIO), Wavre, Belgium.
[4]Institute of Microbiology and Infection, University of Birmingham, Edgbaston, Birmingham, B15 2TT, United Kingdom.

[§]*These authors contributed equally*
[*]Corresponding authors: Joan A. Geoghegan (j.geoghegan@bham.ac.uk), Yves F. Dufrêne (yves.dufrene@uclouvain.be)

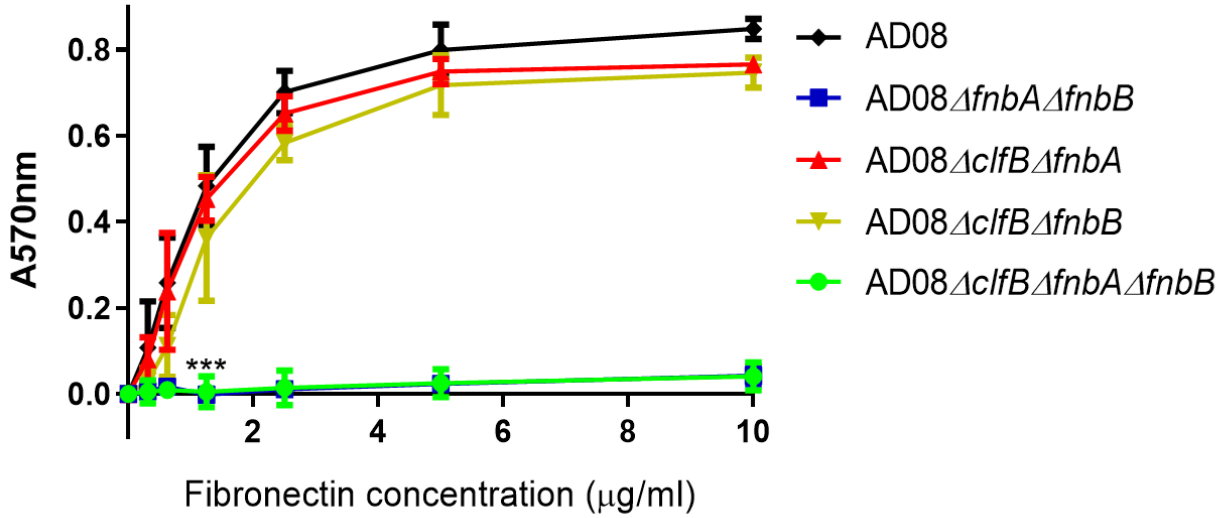

Supplementary Figure 1. Adherence of AD08 mutants to fibronectin.

AD08 or mutants deficient in ClfB and/or FnBPA and/or FnBPB were grown to exponential phase (OD_{600} = 0.35), adjusted to an OD_{600} of 1.0 and incubated in microtiter plates coated with fibronectin. Following incubation, the wells were washed, adherent cells were stained with crystal violet, and the absorbance was read at 570 nm. The datum points on the graph represent the mean values of three independent biological experiments and error bars show the standard deviation. Statistical analysis was performed using a two-way ANOVA with a Dunnett´s multiple comparison test to compare variances between AD08 and the mutants at the 1.25 µg/mL fibronectin concentration. No symbol indicates $P > 0.05$. ***, $P = 0.0001$.

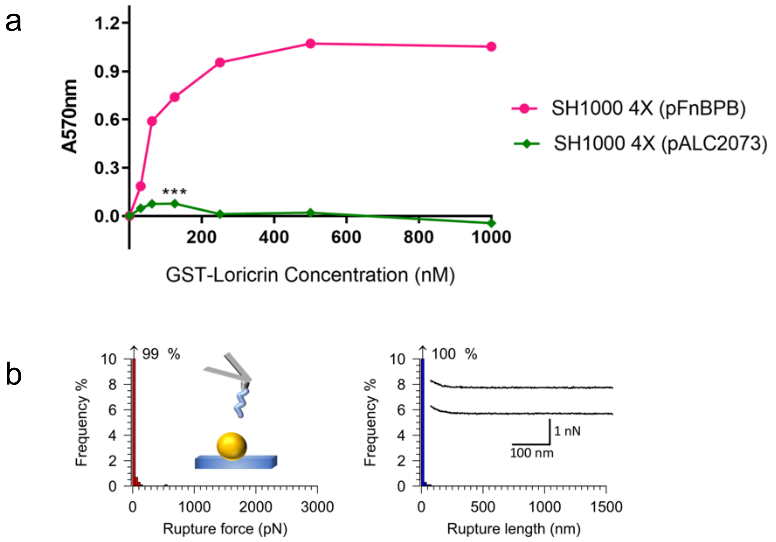

Supplementary Figure 2. *S. aureus* SH1000 deficient in ClfA, ClfB, FnBPA and FnBPB does not bind to loricrin. a) SH1000 and the mutant deficient in ClfA, ClfB, FnBPA and FnBPB (SH1000 4X) were grown to exponential phase (OD_{600} = 0.35), adjusted to an OD_{600} of 1.0 and incubated in microtiter plates coated with GST-loricrin. Following incubation, the wells were washed, adherent cells were stained with crystal violet, and the absorbance was read at 570 nm. The graph shows results from a single experiment that is representative of three independent experiments. The ligand concentration given on the x-axis is the concentration of the solution used to coat the wells. Statistical analysis was performed using a two-way ANOVA with a Sidak´s multiple comparison test to compare variances between the wild type and the mutant at the 125 nM loricrin concentration. ***, $P = 0.00078$. **b)** AFM tips modified with loricrin were used to probe single exponentially grown (OD_{600} = 0.35) staphylococcal cells. Data for one representative *S. aureus* SH1000 4X cell showing the absence of interaction with loricrin. Left. Histogram plot of rupture forces. Right. Histogram plot of rupture lengths with inset showing three representative curves.

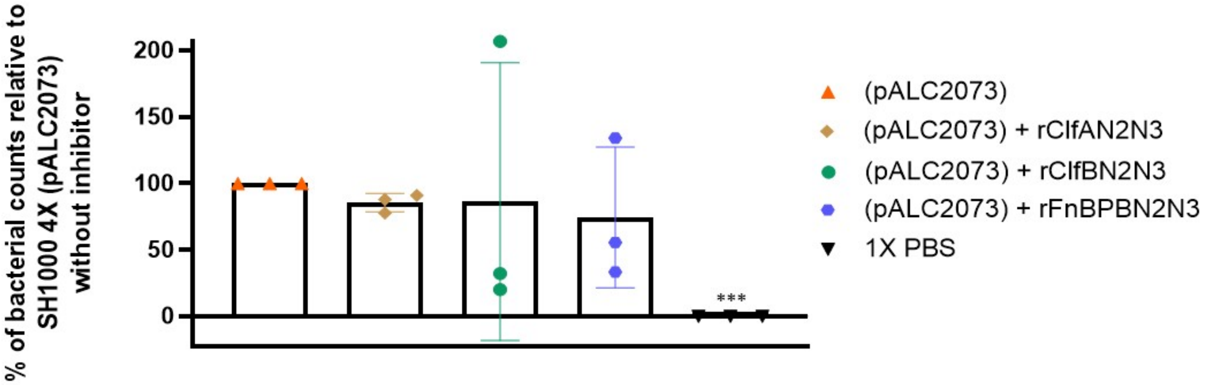

Supplementary Figure 3. Recombinant proteins do not affect *S. aureus* SH1000 4X (pALC2073) adherence to healthy human skin corneocytes. Bacteria were grown in TSB to exponential phase (OD_{600}= 0.35), adjusted to an OD_{600} = 1.0 and incubated with tape strips containing corneocytes previously treated with rClfAN2N3 or rClfBN2N3 or rFnBPBN2N3. Each datum point is from a different biological replicate, and the bars represent the means of three independent experiments, with error bars showing the standard deviation. A no bacteria control was used where PBS alone was incubated with the corneocytes. Tape strips were washed, and adherent bacteria were stained with crystal violet. The number of bacteria cells adhering to 10 corneocytes was counted and the percentage of adherence is shown as comparison with total adherence of SH1000 4X (pFnBPB) to corneocytes that were not pre-treated with recombinant protein. Statistical analysis was performed using a one-way ANOVA with a Dunnett´s multiple comparison test to compare variances between *S. aureus* SH1000 4X (pALC2073) adhering to corneocytes without recombinant protein to the *S. aureus* SH1000 4X (pALC2073) adhering to corneocytes treated with recombinant protein, and the no bacteria control. ***, P = 0.000091.

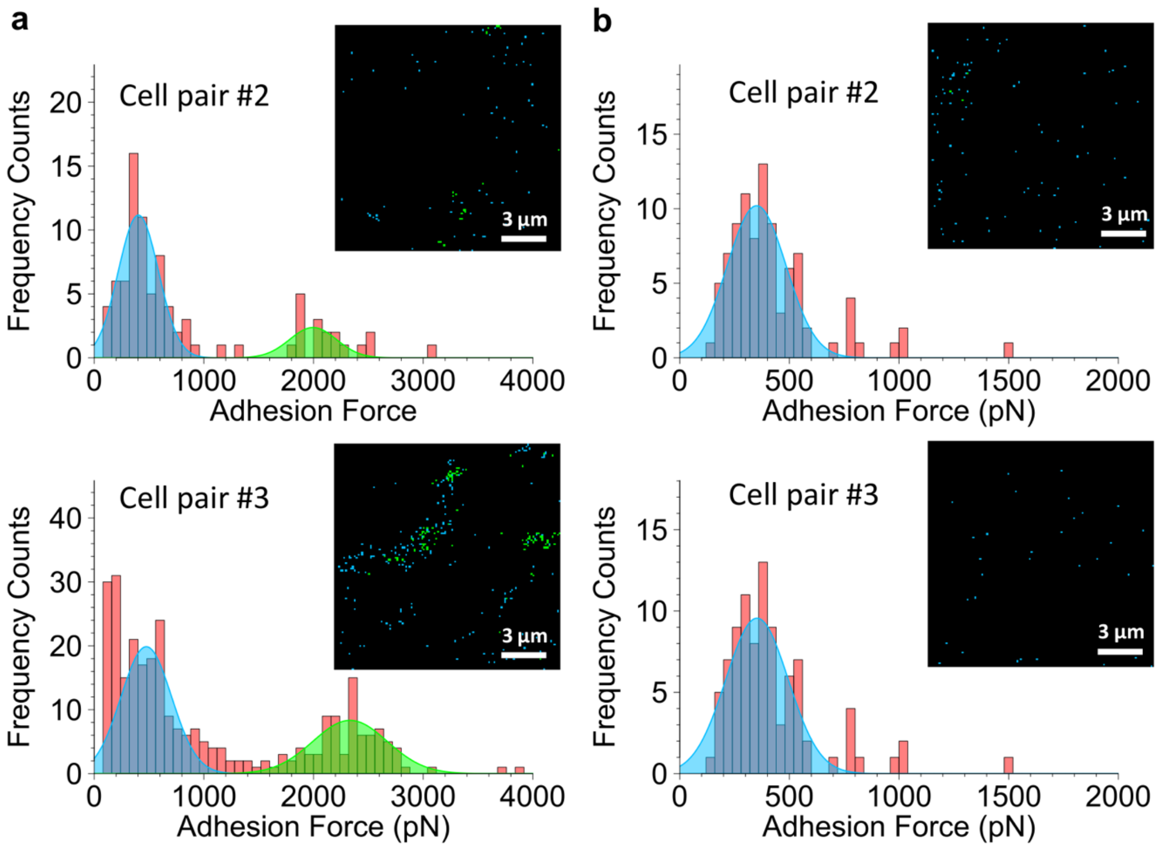

Supplementary Figure 4. FnBPB expressed on living *S. aureus* cells forms ultrastrong single molecular complexes with ligands exposed on corneocyte surfaces. (a) Data for two more *S. aureus* SH1000 4X (pFnBPB) cells used to probe corneocytes in quantitative imaging mode. Histograms of binding forces with inset of the corresponding adhesion map. Black pixels show where zero adhesion was measured, blue pixels show where adhesive interactions between 0 and 1,000 pN were detected. Green pixels are where adhesive interactions greater than 1,000 pN were detected. (b) Data obtained when two more control cells that do not express FnBPB [*S. aureus* SH1000 4X (pALC2073)] were used to probe corneocytes.

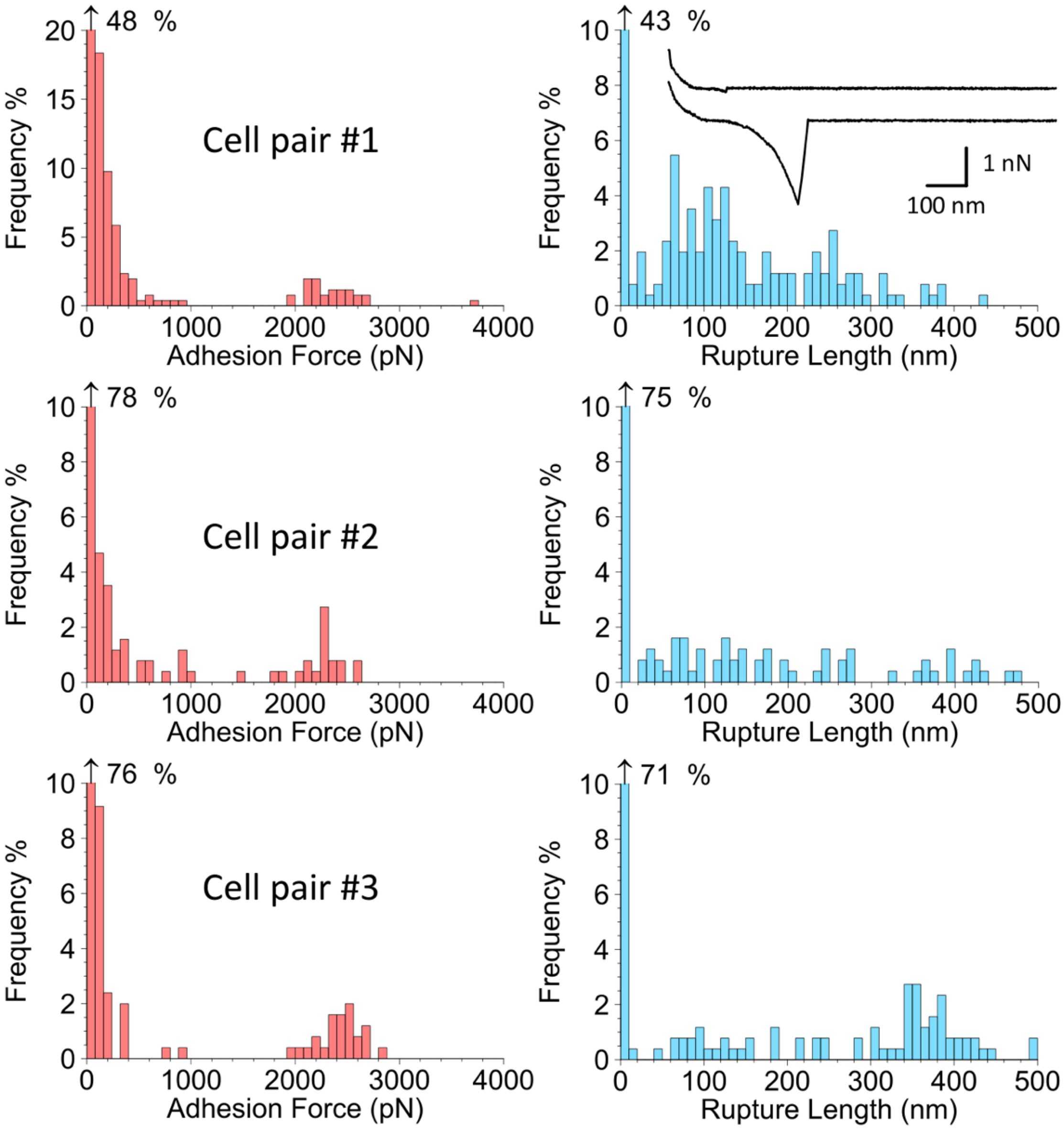

Supplementary Figure 5. Low retraction-speed (1 µm.s^{-1}) force volume mode recordings using *S. aureus* SH1000 4X (pFnBPB) cell probes to probe corneocytes. A force volume map was recorded on a 2 × 2 µm area (16 × 16 pixels) of a corneocyte where ultrastrong binding (> 2,000 pN) was previously detected in QI mode (for cell pair 1, see Figure 7b; and for cell pairs 2 and 3, see Supplementary Figure 4a). Shown are adhesion force (left) and rupture length (right) histograms. The inset shows two representative retraction profiles.

Supplementary Table 1. Bacterial strains.

Strain	Description	Reference
Topp3	*E. coli* strain deficient in proteases for expression of recombinant proteins	Stratagene
XL-1 Blue	*E. coli* strain for deficient in proteases expression of recombinant proteins	Stratagene
IM01B	*E. coli* strain for plasmid propagation. SA08BΩPN25-hsdS	1
AD08	*S. aureus* atopic dermatitis clinical isolate	2
AD08*ΔclfBΔfnbA*	AD08 deficient in *clfB* and *fnbA*	3
AD08*ΔclfBΔfnbB*	AD08 deficient in *clfB* and *fnbB*	3
AD08*ΔfnbAΔfnbB*	AD08 deficient in *fnbA* and *fnbB*	3
AD08*ΔclfBΔfnbAΔfnbB*	AD08 deficient in *clfB* and *fnbA* and *fnbB*	3
SH1000	*S. aureus* strain 8325-4 derivative with a restored *rsbU* gene	4
SH1000 4X	SH1000 deficient in *clfA* and *clfB* and *fnbA* and *fnbB*	5

Supplementary Table 2. Plasmids

**Plasmid**	**Description**	**Reference**
pGEX-4T2	*E. coli* vector for the expression of glutathione S-transferase. Amp^{r}.	GE Lifesciences
pGEX-4T2::HLor	pGEX-4T2 carrying the full-length cDNA encoding human loricrin. Amp^{r}.	6
pQE30::rClfB$_{201–542}$	pQE30 carrying the His-tagged N2 N3 recombinant ClfB. Amp^{r}.	6
pQE30::rFnBPB$_{162-480}$	pQE30 carrying the His-tagged N2 N3 recombinant FnBPB. Amp^{r}.	7
pQE30::rClfA$_{221–559}$	pQE30 carrying the His-tagged N2 N3 recombinant FnBPB. Amp^{r}.	8
pALC2073	Tetracycline inducible expression plasmid. Cm^{r}.	9
pFnBPB	pALC2073 carrying the *fnbB* gene. Cm^{r}.	3

SUPPLEMENTARY REFERENCES

1. Monk, I. R., Tree, J. J., Howden, B. P., Stinear, T. P. & Foster, T. J. Complete bypass of restriction systems for major *Staphylococcus aureus* lineages. *mBio* **6**, e00308-15 (2015).
2. Fleury, O. M. *et al.* Clumping factor B promotes adherence of *Staphylococcus aureus* to corneocytes in atopic dermatitis. *Infect. Immun.* **85**, e00994-16 (2017).
3. Towell, A. M. *et al. Staphylococcus aureus* binds to the N-terminal region of corneodesmosin to adhere to the stratum corneum in atopic dermatitis. *Proc. Natl. Acad. Sci. U. S. A.* **118**, e2014444118 (2021).
4. Horsburgh, M. J. *et al.* σ^B modulates virulence determinant expression and stress resistance: characterization of a functional *rsbU* strain derived from *Staphylococcus aureus* 8325-4. *J. Bacteriol.* **184**, 5457–5467 (2002).
5. O'Neill, E. *et al.* A novel *Staphylococcus aureus* biofilm phenotype mediated by the fibronectin-binding proteins, FnBPA and FnBPB. *J. Bacteriol.* **190**, 3835–3850 (2008).
6. Mulcahy, M. E. *et al.* Nasal colonisation by *Staphylococcus aureus* depends upon clumping factor B binding to the squamous epithelial cell envelope protein loricrin. *PLoS Pathog.* **8**, e1003092 (2012).
7. Burke, F. M., McCormack, N., Rindi, S., Speziale, P. & Foster, T. J. Fibronectin-binding protein B variation in *Staphylococcus aureus*. *BMC Microbiol.* **10**, (2010).
8. O'Connell, D. P. *et al.* The fibrinogen-binding MSCRAMM (clumping factor) of *Staphylococcus aureus* has a Ca2+-dependent inhibitory site. *J. Biolog. Chem.* **273**, 6821–6829 (1998).
9. Bateman, B. T., Donegan, N. P., Jarry, T. M., Palma, M. & Cheung, A. L. Evaluation of a Tetracycline-Inducible Promoter in *Staphylococcus aureus* In Vitro and In Vivo and Its Application in Demonstrating the Role of *sigB* in Microcolony Formation. *Infect. Immun.* **69**, 7851–7857 (2001).
